# *Alu* elements: at the crossroads between disease and evolution

**DOI:** 10.1042/BST20130157

**Published:** 2013-11-20

**Authors:** Jernej Ule

**Affiliations:** *MRC Laboratory of Molecular Biology, Francis Crick Avenue, Cambridge Biomedical Campus, Cambridge CB2 0QH, U.K.; †Department of Molecular Neuroscience, UCL Institute of Neurology, Queen Square, London WC1N 3BG, U.K.

**Keywords:** alternative splicing, *Alu* element, disease, evolution, intron, transposable element, hnRNP, heterogeneous nuclear ribonucleoprotein, iCLIP, individual nucleotide resolution cross-linking and immunoprecipitation, PTS, 6-pyruvoyltetrahydropterin synthase, RBP, RNA-binding protein

## Abstract

The cost of DNA sequencing is decreasing year by year, and the era of personalized medicine and the $1000 genome seems to be just around the corner. In order to link genetic variation to gene function, however, we need to learn more about the function of the non-coding genomic elements. The advance of high-throughput sequencing enabled rapid progress in mapping the functional elements in our genome. In the present article, I discuss how intronic mutations acting at *Alu* elements enable formation of new exons. I review the mutations that cause disease when promoting a major increase in the inclusion of *Alu* exon into mature transcripts. Moreover, I present the mechanism that represses such a major inclusion of *Alu* exons and instead enables a gradual evolution of *Alu* elements into new exons.

## The regulatory arsenal of the non-coding genome

The non-coding sequence (i.e. the sequence that does not code for proteins) represents 98% of our genome. The primary function of this enormous portion of our genome is to contain a vast resource of regulatory elements that control gene expression. This includes the promoter and enhancer elements for transcriptional regulation, and a variety of diverse elements that are needed for post-transcriptional regulation of gene expression. This second class of regulatory elements is located mainly in introns of pre-mRNAs or in the untranslated regions of mRNAs, where it controls the diverse post-transcriptional processes, including RNA editing or modification (such as methylation), alternative splicing, 3′ end processing, mRNA localization, translation and decay. Together, the arsenal of regulatory elements within the non-coding portion of the genome allows the fine-balanced gene expression appropriate to each cell type in our body, and enables these cells to respond appropriately to the diverse developmental and environmental stimuli.

## The evolutionary potential hidden within introns

Introns represent 23% of the sequence of human genome. Many important non-coding RNAs, such as miRNAs and snoRNAs (small nucleolar RNAs), are expressed from introns. Moreover, introns are a rich resource of regulatory elements, which can bind to diverse RBPs (RNA-binding proteins). Many diverse RBPs bind these intronic elements, most often to repress recognition of nearby splice sites [1]. These elements regulate splicing of alternative exons, but they are also important to repress splicing from the vast array of cryptic splicing elements that are dispersed throughout introns. The cryptic splicing elements are in a poised state: they do not normally contribute to splicing, but even subtle mutations can convert them into active splicing elements. Interestingly, one of the primary sources of cryptic splicing elements are transposable elements [2].

## The role of transposable elements in gene regulation

Some 42% of the human genome is made of transposable elements [3]. These elements have profound effects on genome structure and function. They can mediate structural changes, for instance by promoting DNA recombination. Moreover, they can affect expression of nearby genes in numerous ways, by acting as transcriptional enhancers or silencers, as regulators of translation, or as a source of new sites for pre-mRNA processing. Their evolutionary importance is clear from the finding that transposable elements reshaped the human transcriptional landscape by contributing hundreds of thousands of novel regulatory elements to the primate lineage [3,4]. In the present article, I review the importance of *Alu* elements, the most common transposable element in the primate genomes, in the formation of new exons.

## *Alu* elements as a source of new exons

*Alu* elements are retrotransposable elements that have originated from the small cytoplasmic 7SL RNA. The event when a copy of the 7SL RNA became a precursor of the *Alu* elements took place early in the evolution of supraprimates [5]. *Alu* elements occur in large numbers in primate genomes and comprise over 10% of the human genome. Over 650000 *Alu* elements reside in the introns of protein-coding genes. When inserted in an antisense orientation, an *Alu* element contains several cryptic splice sites, and is therefore an excellent template for the production of new exons. Thus the human genome contains a large reservoir of *Alu* elements that can turn into exons. Indeed, approximately 5% of internal alternative exons in the human transcriptome originate from *Alu* elements [6].

Importantly, some of these exonized *Alu* elements are unique to the human genome, or are spliced in a tissue-specific manner only in humans. For instance, an *Alu*-derived exon in the *SEPN1* (selenoprotein N1) gene is under tissue-specific control in humans. In contrast with chimpanzees, where it is a minor mRNA isoform in all tissues, the *Alu*-derived *SEPN1* exon is part of the dominant mRNA isoform in human muscle tissue [7]. It is clear that exonization of *Alu* elements played an important role in primate evolution [2].

## Controlling the emergence of *Alu* exons

When they initially turn into exons, *Alu* elements generally introduce premature stop codons, leading to mRNA degradation or aberrant proteins [8]. Thus, if new *Alu* elements became part of the dominant mRNA isoform, they would almost invariably be deleterious for the cell. It is therefore not surprising that our cells employ a specific RBP to repress aberrant incorporation of deleterious *Alu* exons into mature transcripts.

The mechanism protecting the transcriptome from aberrant exonization of *Alu* elements was revealed by the iCLIP (individual nucleotide resolution cross-linking and immunoprecipitation) technique, which documents the protein–RNA interactions that take place inside living cells [9]. Quantitative analysis of genome-wide binding profiles revealed that two RBPs compete for binding to the antisense *Alu* elements, and thereby control the emergence of new *Alu* exons. The first of these proteins is hnRNP (heterogeneous nuclear ribonucleoprotein) C, an abundant nuclear RBP that binds to uridine tracts that are present within the antisense *Alu* elements. A quarter of hnRNP C binding maps to *Alu* elements. Strikingly, depletion of hnRNP C leads to aberrant exonizations of *Alu* elements in over 1000 transcripts [8].

The explosion of new *Alu* exons upon depletion of hnRNP C revealed that thousands of antisense *Alu* elements are in a poised state, such that removal of a single RBP is sufficient for exonization. How does hnRNP C manage to keep a lid on the steaming pot of *Alu* elements? The answer was provided by a quantitative iCLIP analysis of U2AF65, an RBP that binds to 3′ splice sites and thereby initiates the splicing process. Analysis of the genome-wide U2AF65-binding profile demonstrated that U2AF65 gains access to the uridine tracts within antisense *Alu* elements upon removal of hnRNP C from the cells. Thus hnRNP C acts by competing with U2AF65 for binding to the antisense *Alu* elements, and thereby prevents the recognition of cryptic 3′ splice sites [8].

## *Alu* exons in disease

Activation of cryptic exons can lead to diverse human genetic diseases. Approximately 25% of these are caused by aberrant activation of cryptic exons from antisense *Alu* elements [10]. Mutations that remove hnRNP C-dependent repression can cause the activation of *Alu* exons. An example of this process is evident in the mutation within the *Alu* element of the *PTS* (6-pyruvoyltetrahydropterin synthase) gene. PTS is required for the biosynthesis of tetrahydrobiopterin, also known as BH_4_, an essential cofactor and regulator of enzymes involved in serotonin biosynthesis and nitric oxide synthase activity. Mutations in *PTS* result in hyperphenylalaninaemia [11].

**Figure 1:**
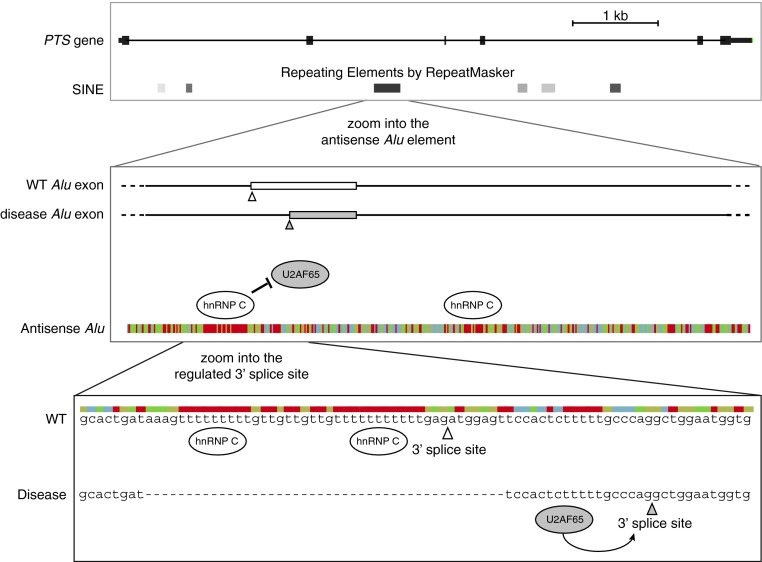
hnRNP C repression of *Alu* exonization in the *PTS* gene is relevant for disease (**A**) A view of the exon/intron structure of the *PTS* gene. The boxes below show the positions of short interspersed elements (SINE), which include *Alu* elements, as defined by RepeatMasker. (**B**) The disease-relevant *Alu* element within the *PTS* gene is shown at greater resolution. The position of the two exons that can emerge from this *Alu* element are schematically indicated: the blank exon is rarely included in the wild-type (WT) cells, whereas the grey exon is part of the dominant isoform in disease. Below, the sequence is shown in a colour-coded fashion. The uridine tracts (in red) bind to hnRNP C, which represses binding of U2AF65. (**C**) The two 3′ splice sites that can lead to the formation of *Alu* exon are shown at nucleotide resolution. In wild-type cells, hnRNP C binds to the long uridine tracts to repress the binding of U2AF65, and therefore the 3′ splice site marked by the open arrowhead is rarely used. In disease, the primary hnRNP C-binding site is deleted, and therefore U2AF65 can bind to the pyrimidine tract upstream of the 3′ splice site that is marked by the grey arrowhead. This leads to strong inclusion of the grey exon that is shown in (**B**) [8].

Splicing reporter assays demonstrated that one of the disease-causing mutations removes the uridine tract from *Alu* elements, which is required for hnRNP C binding. This allows binding of U2AF65 to the *Alu* element, leading to activation of a new 3′ splice site, and a deleterious *Alu* exon that becomes part of the dominant transcript (Figure 1). The resulting transcript contains a premature stop codon, and is therefore targeted for degradation, whereas the original isoform required for production of the full-length protein is lost [8].

## Evolution: the path trodden with baby steps

Without hnRNP C binding, many intronic *Alu* elements immediately turn into exons and thereby severely damage the resident gene. Therefore mutations can convert an *Alu* element into a dominant exon when completely removing hnRNP C binding, which can lead to disease. However, this is just an extreme case in the diverse spectrum of mutations that can modulate the formation of *Alu* exons. As demonstrated by the splicing reporter assays, point mutations within uridine tracts modify the competitive binding between hnRNP C and U2AF65 in a subtle way, and thereby lead to only a minor change in the exonization of the corresponding *Alu* element [8]. Thus the competitive binding of hnRNP C and U2AF65 can be modulated via sequential mutations, which gradually increase the binding of U2AF65.

The hnRNP C/U2AF65 regulatory mechanism ensures that evolution of new exons from *Alu* elements occurs via an incremental process. Mutations can promote formation of *Alu* exons either by modifying the competitive binding of U2AF65 and hnRNP C, or by increasing the strength of splice sites within *Alu* elements, or act on additional enhancer or silencer elements [2]. A subtle increase in the inclusion of *Alu* exon does not perturb the expression of the normal mRNA isoform, therefore there is little negative selection pressure to remove the new mRNA isoform containing the *Alu* exon. This enables additional mutations to accumulate within the *Alu* exon itself, which can remove the premature stop codons and change the coding potential of the exon. Each mutation can thus make a small step towards ultimately creating an *Alu* exon with a novel and beneficial function for the cell [8]. Thus hnRNP C enables exonization of *Alu* exons to proceed in small steps, without hampering the expression of the primary mRNA isoform. In other words, evolution treads the path of *Alu* exons with baby steps in order to minimize the chance that a new *Alu* exon would cause disease.

## Conclusion

The beauty of evolution is in its ability to fine-tune the cellular function in the face of changing environment, without thereby damaging the core cellular mechanisms. The carefully regulated exonization of *Alu* exons enables the evolutionary path to be taken in small reversible steps. The formation of *Alu* exons thus provides an example of how the fine-balanced mechanisms can act to minimize the chance that disease would occur as a side effect of evolutionary processes. Nevertheless, the core mechanisms in our cells are very complex, therefore it is inevitable that the evolutionary process will occasionally make a step too large, and disrupt the primary mRNA isoform in a manner that will cause disease.

*Alu* elements are one of the many different types of elements hidden within introns, which enable evolution to seek new solutions by creating alternative mRNA isoforms without destroying the primary mRNA isoform. It is most likely that many other RBPs play a role similar to hnRNP C, ensuring that creation of new exons follows a gradual process, instead of a sudden activation of cryptic exons that is likely to cause genetic disease. Further application of genomic technologies and new computational tools will facilitate the discovery of the intronic hotspots where mutations can lead either to disease or to the evolution of new exons.

Apart from producing new exons, transposable elements can modulate the transcriptome in additional ways. In DNA, transposable elements can interact with DNA-binding proteins to change the transcription of nearby genes, and in RNA, they can change the structure of RNA, influence 3′ end processing, or mRNA localization or translation [3]. Similar to the hnRNP C-dependent repression of *Alu* exons, cells contain diverse mechanisms to buffer the uncontrolled effects of transposable elements on transcriptome. This includes DNA methylation, RNA editing, nuclear mRNA retention and RNAi-based silencing [12]. All of these mechanisms may help to minimize the chance that transposable elements would cause disease, but would instead enable evolutionary exploration of changes in gene expression and new cellular functions. Increased understanding of the interplay between the transposable elements and the regulatory machinery of the cell will help us to better interpret the functional impact of SNPs (single nucleotide polymorphisms) and other types of variation within the non-coding portion of our genome [13].
